# Retraction: Effect of potassium fertilizer on the growth, physiological parameters, and water status of *Brassica juncea* cultivars under different irrigation regimes

**DOI:** 10.1371/journal.pone.0294881

**Published:** 2023-11-16

**Authors:** The PLOS ONE Editors

This article [1] was identified as being linked to a series of submissions for which PLOS noted concerns about authorship and peer review.

In addition, concerns were raised about the size of the error bars presented for each result in Figs 1 and 2. Primary data were not provided with the article, contrary to the Data Availability statement.

The authors provided individual level data underlying the published results. However, editorial assessment of these data raised further concerns regarding the validity, reliability, and integrity of these data.

The *PLOS ONE* Editors retract this article due to the unresolved concerns. We regret that the issues were not addressed prior to the article’s publication.

PK did not agree with the retraction. PR, IS, NS, LW, AAB, HD, and AN either did not respond directly or could not be reached.
